# Compared planning dosimetry of TOMO, VMAT and IMRT in rectal cancer with different simulated positions

**DOI:** 10.18632/oncotarget.14923

**Published:** 2017-01-31

**Authors:** Jang-Chun Lin, Jo-Ting Tsai, Li-Jhen Chen, Ming-Hsien Li, Wei-Hsiu Liu

**Affiliations:** ^1^ Department of Radiation Oncology, Shuang Ho Hospital, Taipei Medical University, Taipei, Taiwan, ROC; ^2^ Department of Radiology, School of Medicine, College of Medicine, Taipei Medical University, Taipei, Taiwan, ROC; ^3^ Graduate Institute of Clinical Medicine, College of Medicine, Taipei Medical University, Taipei, Taiwan, ROC; ^4^ Department of Radiation Oncology, Tri-Service General Hospital, National Defense Medical Center, Taipei, Taiwan, ROC; ^5^ Department of Neurological Surgery, Tri-Service General Hospital and National Defense Medical Center, Taipei, Taiwan, ROC; ^6^ Graduate Institute of Medical Sciences, National Defense Medical Center, Taipei, Taiwan, ROC

**Keywords:** rectal cancer (RC), helical tomotherapy (TOMO), volumetric modulated arc therapy (VMAT), intensity modulated radiotherapy (IMRT), radiation dosimetry

## Abstract

**Objectives:**

To compare treatment plans for helical tomotherapy (TOMO), volumetric modulated arc therapy (VMAT) and intensity-modulated radiotherapy (IMRT) for locally advanced rectal cancer (LARC).

**Materials and Methods:**

This retrospective study from December 2010 to June 2013 included 20 patients with LARC who received neoadjuvant concurrent chemoradiotherapy (CCRT) with radiation doses of greater than 50.4 Gy. Dosimetric quality was evaluated based on doses to organs at risk (OARs), including small bowel, urinary bladder and bilateral femoral head, over the same coverage of the clinical target volume (CTV).

**Results:**

In supine comparison of IMRT with VMAT, VMAT treatment plan had a lower hot spot dose (p=0.0154) and better conformity index (CI, p=0.0036) and homogeneity index (HI, p=0.0246). Lower bladder V34.98 (p=0.0008), V40 (p=0.0058), mean dose (p<0.0001), femoral head mean dose (p=0.0089), V30 (p<0.0001), V40 (p=0.0013) and better CI (p<0.0001) and HI (p=0.0001) were observed for TOMO compared with IMRT. Patients with LARC receiving TOMO planning had lower bladder V34.98 (p=0.0021), V40 (p=0.0055), mean dose (p=0.0039), femoral head mean dose (p=0.0060), V30 (p<0.0001), and V40 (p=0.0044) and better CI (p=0.0157) and HI (p=0.0292) than VMAT. Comparing prone and supine position image planning, there were no significant differences, including in OARs in the three planning systems, except for lower bladder V34.98 (p=0.0403) in the supine position using TOMO.

**Conclusions:**

Using modern radiation techniques, neither prone nor supine positions provide better values for OARs. TOMO was superior to IMRT and VMAT in sparing OARs and planning quality parameters.

## INTRODUCTION

In Taiwan, preoperative chemoradiotherapy (CCRT) has become a widely accepted treatment modality for locally advanced rectal cancer (LARC). Other groups have revealed that preoperative CCRT is associated with significantly less acute and chronic toxicity, a lower local recurrence rate and a higher organ preservation rate than postoperative CCRT [1]. In a previous study, preoperative CCRT resulted in a significant improvement of the 5-year disease-free survival rate [2].

Thus, LARC radiotherapy is a complex problem because of the shapes of the target volumes and the need to minimize the involvement of organs at risk (OARs), such as the bladder, small bowel and femoral heads [3]. In general, patients with rectal carcinoma should be treated in the prone position to reduce the volume of the small bowel within the pelvis [4]. Due to the use of modern RT techniques, patients with LARC can be maneuvered to decrease the volume of the small bowel in the radiation field and to treat with a full bladder in the supine position with the use of bowel-displacement tools such as a foam board mound designed to push the full bladder posteriorly and cephalad. Different positions of computed tomography (CT) simulation might be able to reduce the radiation dose of the small bowel. Acute and chronic small bowel and rectal toxicities may limit further dose escalation or lead to premature termination of the radiation course, potentially decreasing therapy effectiveness.

Several studies have observed a strong dose–volume relationship in the development of acute small bowel toxicity in patients receiving preoperative CCRT [5]. Consequently, the application of highly conformal treatment modalities, such as helical tomotherapy (TOMO), intensity-modulated radiation therapy (IMRT) and volumetric modulated arc therapy (VMAT), has been of great interest for producing highly conformal dose distributions in the target volumes and minimizing the dose to OARs. Several planning studies have been performed for LARC using different treatment approaches, such as VMAT, IMRT and three-dimensional conformal radiotherapy (3D-CRT) [6–8]. However, several studies have revealed advantages of IMRT over 3D-CRT in target coverage and normal tissue sparing for rectal cancer patients; drawbacks of the IMRT technique have also been reported [7, 9]. VMAT can achieve a high quality of conformal dose distributions and is essentially an alternative form of IMRT; in addition, the improvement in radiation delivery efficiency and the reduction in MU usage can be overcome by VMAT [10, 11]. Most of the planning studies in various tumor sites have compared VMAT with either fixed-field IMRT or 3D-CRT techniques; moreover, in rectal cancer, VMAT has clear superiority over 3D-CRT with regard to improving dosimetric parameters and sparing OARs [8, 12]. Furthermore, a few studies have revealed dosimetric differences among these radiation techniques in the treatment of simultaneous integrated boost radiotherapy for preoperative LARC, revealing only that VMAT planning system can provide similar sparing of OARs as IMRT, with higher efficiency [13]. However, the dose distinction between TOMO and VMAT is not well documented. The present study is the first planning study to compare TOMO, IMRT and VMAT in rectal cancer, including comparing the dosimetric parameters among TOMO, VMAT and conventional IMRT for patients with rectal cancer and evaluating the dose distributions, planning target volumes (PTVs) and OARs

## RESULTS

### Patient characteristics

Twenty patients (17 males and 3 females) previously treated at our facility were selected for 15 supine simulated positions and 5 prone simulated position. All patients were diagnosed with moderately to poorly differentiated adenocarcinoma of the rectum or rectosigmoid junction. Nine patients were stage II, 2 were stage IIIA, 2 were stage IIIB, and 7 were stage IIIC according to the 6th edition of the AJCC, 2006 Criteria, and the 7th edition of the AJCC, 2010 Criteria. All patients in this study received preoperative concurrent chemotherapy. Table 1 summarizes the patients’ characteristics.

**Table 1:** Patients and tumor characteristics (N=20)

Patient characteristic		Supine (n=15)	Prone (n=5)
		N (%)	N (%)
**Gender**	Male	13(86.7)	4(80)
	Female	2(13.3)	1(20)
**Age (year-old)**	>60	6(40)	3(60)
	<60	9(60)	2(40)
	Median	59	64
	Range	40–78	51–71
**ECOG**	(0/1)	12/3(80/20)	3/2(60/40)
**Tumor location**	(Rectum/R-S junction)	12/3(80/20)	4/1(80/20)
**Tobacco use**	( +/− )	3/12(20/80)	0/5(0/100)
**Alcoholic drinking**	( +/− )	0/15(0/100)	0/5(0/100)
**AJCC Stage**	II/IIIA/IIIB/IIIC	8/1/2/4(53.3/6.7/13.3/26.7)	1/1/0/3(20/20/0/60)
**Concurrent therapy**	No concurrent Tx. / chemotherapy	6/14(30/70)	12/9(57.1/42.9)

R-S: recto-sigmoid; Tx. : treatment

### IMRT vs. VMAT

Planning dosimetry in the supine simulated position of the 15 patients receiving RT compared IMRT with VMAT; the VMAT treatment plan had a lower hot spot dose (p=0.0154) and better conformity index (CI, p=0.0036) and homogeneity index (HI, p=0.0246). Both techniques had similar coverage of the PTVs (p=0.4214). In the prone simulated position, only PTV coverage in VMAT planning was significant better than IMRT planning (p=0.0334). There were no differences in OARs between IMRT and VMAT. Table 2A summarizes the IMRT, VMAT and TOMO planning dosimetric parameters and OAR results in detail.

**Table 2A:** Dosimetric results of IMRT, VMAT and TOMO for planning dosimetric parameters and organs at risk

Variable	IMRT		VMAT		TOMO	
	Mean ± SD					
	Supine	Prone	Supine	Prone	Supine	Prone
**D_97_ (PTV_48.89_)**	98.62±1.12	98.39±0.95	99.02±1.16	99.47±0.60	98.94±1.03	99.21±0.71
**Hot spot(Gy)**	53.95±0.78	53.52±0.93	53.33±0.70	53.84±0.90	49.76±13.65	53.50±0.76
**CI**	0.81±0.04	0.76±0.08	0.85±0.04	0.80±0.08	0.89±0.05	0.88±0.13
**HI**	1.07±0.02	1.06±0.02	1.06±0.02	1.05±0.01	1.04±0.02	1.05±0.01
**Small bowelDmax (Gy)**	45.32±12.04	41.49±9.88	45.16±12.04	41.54±10.53	44.70±11.84	39.81±9.08
**BladderDmax (Gy)**	50.39±1.95	50.00±1.27	50.59±2.49	50.16±4.43	48.36±4.71	50.00±3.47
**Mean dose (Gy)**	29.30±5.75	32.35±5.58	26.66±6.77	26.77±6.78	21.10±5.68	22.24±9.63
**V_30_ (%)**	45.81±23.42	57.41±31.58	39.19±19.73	38.87±25.04	14.27±11.17	31.75±29.50
**V_34.98_ (%)**	28.98±18.67	40.67±25.77	24.45±15.77	28.95±21.85	3.90±5.67	8.03±12.46
**V_40_ (%)**	17.04±11.65	25.37±19.24	17.01±11.95	19.34±19.46	7.07±7.08	13.97±19.18
**V_44.87_ (%)**	5.93±6.14	9.04±7.52	7.53±6.40	9.16±8.18	3.90±5.67	8.03±12.46
**V_48.07_ (%)**	1.70±2.56	2.05±2.69	2.20±2.86	2.70±3.16	1.64±3.77	1.43±2.72
**V_50_ (%)**	0.83±1.72	0.36±0.66	0.71±1.40	0.92±1.58	0.99±2.75	3.32±6.32
**Femoral headMean dose (Gy)**	27.60±3.91	26.05±8.67	28.47±4.25	28.49±4.71	21.38±9.87	17.96±10.20
**V_30_ (%)**	37.10±20.65	37.38±40.78	41.65±31.38	38.55±24.51	10.08±7.44	12.26±10.05
**V_40_ (%)**	3.62±3.36	6.98±14.00	3.77±4.24	5.99±10.52	0.43±0.66	2.29±5.12

D_9_7: the percentage of the prescribed dose covering 97% volume of PTV; IMRT: intensity modulated radiation therapy; PTV_48.89_: planning target volume 48.89 Gy; SD: standard deviation; VMAT: volumetric modulated radiation therapy; TOMO: helical tomotherapy; Vx: the percentage of organ receiving more or equal to x Gy.

### IMRT vs. TOMO

**Figure 1:**
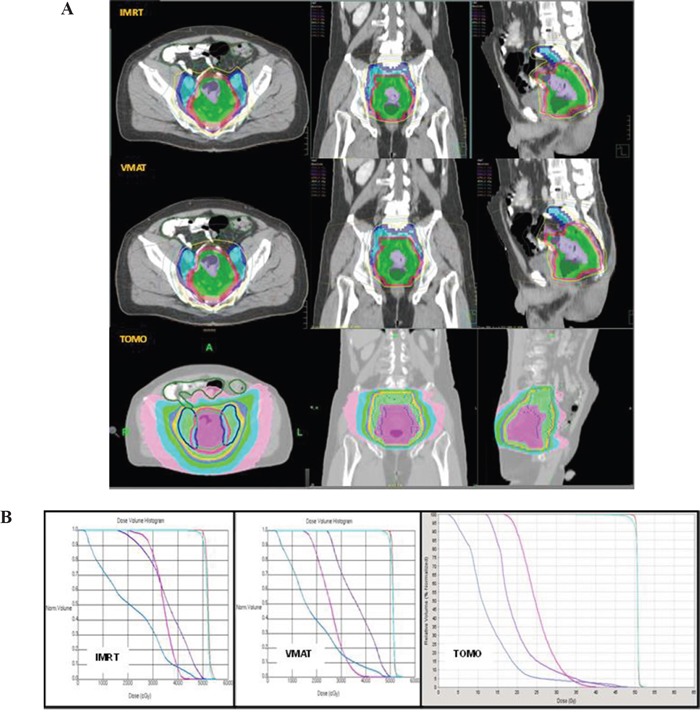
**A**. Isodose curve distribution of IMRT, VMAT, TOMO in a patient with LARC in the supine position. **B**. Depicts the dose-volume histograms (DVHs) of IMRT, VMAT, TOMO in a patient with LARC in the supine position. Blue line: small bowel; Pink line: femoral head; Purple line: bladder; Light blue line: PTV; Orange line: CTV.

In comparing IMRT with TOMO, lower bladder V34.98 (p=0.0008), V40 (p=0.0058), mean dose (p<0.0001), femoral head mean dose (p=0.0089), V30 (p<0.0001), and V40 (p=0.0013) and better CI (p<0.0001) and HI (p=0.0001) were observed in the supine position by TOMO planning. For patients with LARC in a prone simulated position, a lower bladder mean dose (p=0.0427) was observed in the planning by TOMO compared to IMRT. A few OARs and dosimetric parameters, including bladder V30 (p=0.0703), femoral head (p=0.0805), CI (p=0.0578) and PTV coverage (p=0.0808), exhibited a tendency toward more favorable values in TOMO than IMRT. This result might be attributable to the patient sample size, which was too small to allow a significant analytical difference to be observed. Figure 1A and 1B depicts the isodose distribution and dose-volume histograms (DVHs) of IMRT, VMAT and TOMO in a patient with LARC in the supine position.

### VMAT vs. TOMO

**Figure 2:**
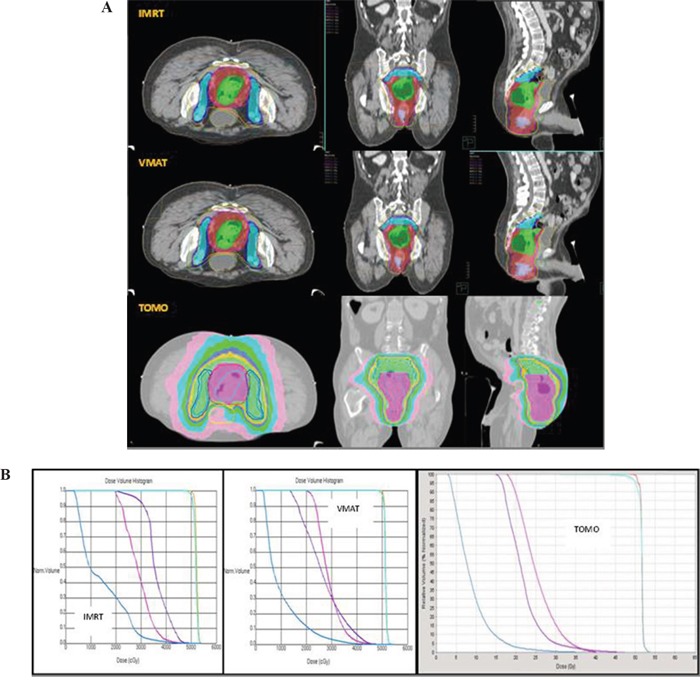
**A**. Isodose curve distribution of IMRT, VMAT, TOMO in a patient with LARC in the prone position. **B**. DVHs of IMRT, VMAT, TOMO in a patient with LARC in the prone position. Blue line: small bowel; Pink line: femoral head; Purple line: bladder; Light blue line: PTV; Orange line: CTV.

Patients with LARC in a supine-simulated position receiving TOMO planning had lower bladder V34.98 (p=0.0021), V40 (p=0.0055), mean dose (p=0.0039), femoral head mean dose (p=0.0060), V30 (p<0.0001), and V40 (p=0.0044) and better CI (p=0.0157) and HI (p=0.0292) than VMAT; bladder Dmax and bladder V44.87 were similar (p=0.0595 and 0.0559) for the 2 techniques. The femoral head mean dose (p=0.0321) in the prone position differed significantly between TOMO and VMAT planning. Other OARs and planning parameters exhibited no apparent difference between TOMO and VMAT. The analytic comparison data for IMRT vs. VMAT, IMRT vs. TOMO, and VMAT vs. TOMO are presented in Table 2B. The isodose distributions and DVHs of IMRT VMAT and TOMO in one patient with LARC in the prone position are displayed in Figure 2A and 2B.

**Table 2B:** Dosimetric results of analytic data comparison for IMRT vs. VMAT, IMRT vs. TOMO, and VMAT vs. TOMO

Variable	IMRT vs. VMAT		IMRT vs. TOMO		VMAT vs. TOMO	
	p value					
	Supine	Prone	Supine	Prone	Supine	Prone
**D_97_ (PTV_48.89_)**	0.1719	0.0334	0.2102	0.0808	0.4214	0.2652
**Hot spot(Gy)**	0.0154	0.2921	0.1273	0.4890	0.1640	0.2654
**CI**	0.0036	0.1851	<0.0001	0.0578	0.0157	0.1683
**HI**	0.0246	0.2759	0.0001	0.1160	0.0292	0.1784
**Small bowelDmax (Gy)**	0.4856	0.4976	0.4444	0.3934	0.4588	0.3946
**BladderDmax (Gy)**	0.4022	0.4712	0.0699	0.4966	0.0595	0.4737
**Mean dose (Gy)**	0.1301	0.0975	<0.0001	0.0427	0.0039	0.2084
**V_30_ (%)**	0.2050	0.1675	0.2435	0.0703	0.2250	0.2405
**V_34.98_ (%)**	0.2395	0.2303	0.0008	0.1212	0.0021	0.2959
**V_40_ (%)**	0.4977	0.3047	0.0058	0.1879	0.0055	0.3239
**V_44.87_ (%)**	0.2449	0.4907	0.1781	0.4406	0.0559	0.4351
**V_48.07_ (%)**	0.3094	0.3665	0.4792	0.3633	0.3251	0.4336
**V_50_ (%)**	0.4150	0.2482	0.4272	0.2182	0.3646	0.3638
**Femoral headMean dose (Gy)**	0.2805	0.3002	0.0089	0.0805	0.0060	0.0321
**V_30_ (%)**	0.1633	0.4873	<0.0001	0.0792	<0.0001	0.0655
**V_40_ (%)**	0.1679	0.4514	0.0013	0.1812	0.0044	0.1757

D_97_: the percentage of the prescribed dose covering 97% volume of PTV; IMRT: intensity modulated radiation therapy; PTV_48.89_: planning target volume 48.89 Gy; SD: standard deviation; VMAT: volumetric modulated radiation therapy; TOMO: helical tomotherapy; Vx: the percentage of organ receiving more or equal to x Gy.

### Supine vs. prone in the IMRT, VMAT and TOMO planning techniques

**Figure 3:**
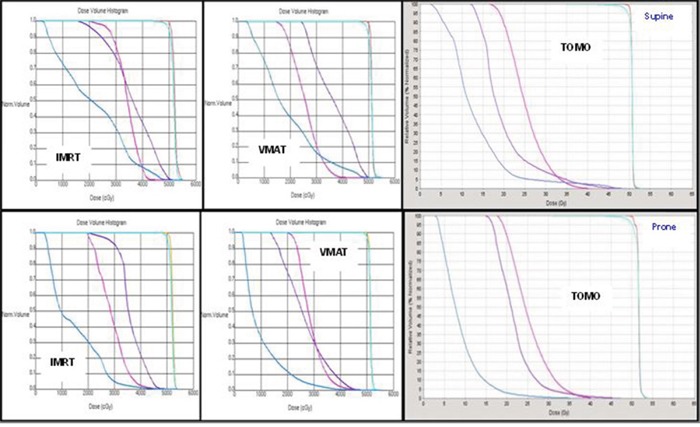
DVHs for the two different simulated positions in a typical case with plans by the three different techniques Blue line: small bowel; Pink line: femoral head; Purple line: bladder; Light blue line: PTV; Orange line: CTV.

Bladder V34.98 (p=0.0734), V40 (p=0.0733), and mean dose (p=0.0689) were similar in the supine-simulated and prone positions in patients with LARC using IMRT; however, IMRT had better planning quality with CI (p=0.0168) in the supine position than in the prone position. There were no differences in OARs in VMAT between the supine and prone positions, but VMAT had a better CI (p=0.0466) and lower hot spot dose (p=0.0392) in the supine position than prone. TOMO planning had lower bladder V34.98 (p=0.0403) and similar femoral head V40 (p=0.0685) in the supine position to that in the prone position. None of the planning quality parameters, including PTV coverage, CI, HI and hot spot, differed significantly in the analysis. All detailed matched-pairs results are summarized in Table 3. Figure 3 displays the DVHs for the two different simulated positions in a typical case with plans by the three different techniques.

**Table 3:** Dosimetric results for planning parameters and comparison for organs at risk in the supine vs. prone simulated position

Variable	Supine	v.s	Prone
	p value		
	IMRT	VMAT	TOMO
**D_97_ (PTV _48.89_)**	0.2628	0.0927	0.2050
**Hot spot(Gy)**	0.0812	0.0392	0.1533
**CI**	0.0168	0.0466	0.4597
**HI**	0.1023	0.3764	0.2108
**Small bowelDmax (Gy)**	0.1682	0.1866	0.1020
**BladderDmax (Gy)**	0.2612	0.3660	0.1390
**Mean dose (Gy)**	0.0689	0.4806	0.2212
**V_30_ (%)**	0.1203	0.4837	0.1921
**V_34.98_ (%)**	0.0734	0.2514	0.0403
**V_40_ (%)**	0.0733	0.3227	0.0893
**V_44.87_ (%)**	0.1025	0.2646	0.1139
**V_48.07_ (%)**	0.3535	0.3170	0.1955
**V_50_ (%)**	0.1655	0.3451	0.3248
**Femoral headMean dose (Gy)**	0.2553	0.4654	0.1687
**V_30_ (%)**	0.3629	0.1664	0.2455
**V_40_ (%)**	0.1727	0.1327	0.0685

D_97_: the percentage of the prescribed dose covering 97% volume of PTV; IMRT: intensity modulated radiation therapy; PTV_48.89_: planning target volume 48.89 Gy; SD: standard deviation; VMAT: volumetric modulated radiation therapy; TOMO: helical tomotherapy; Vx: the percentage of organ receiving more or equal to x Gy.

## DISCUSSION

Our dosimetric study compared the dose variability of the TOMO, VMAT, and IMRT planning methods for patients with LARC post radiotherapy with simultaneously integrated boost planning. Few studies have studied different treatment techniques, such as IMRT, VMAT and proton methods, for rectal cancer [8, 16]. Our study investigated simultaneously integrated radiotherapy in rectal cancer with different simulated positions. A previous prostate cancer-related study [17] revealed different values of dose distribution between the small bowel, bladder, and femoral heads when comparing the supine and prone position. In the present study, there were no differences in the dose distribution between the small bowel, bladder, and femoral head in the supine and prone positions among TOMO, VMAT, and IMRT, except for the bladder V34.98, which was lower in supine using TOMO. Planning quality parameters including PTV coverage, CI, HI and hot spot did not differ significantly between the supine and prone positions using TOMO, VMAT, and IMRT, except for better CI in supine using IMRT or VMAT. In traditional radiotherapy, patients with rectal carcinoma are treated in the prone position to reduce the volume of the small bowel within the pelvis [4], but the use of modern RT techniques would overcome this disadvantage of the supine position.

For the comparisons of TOMO to VMAT, TOMO to IMRT and VMAT to IMRT, our results revealed an obvious superiority of CI and HI. With regard to sparing OARs, the TOMO plan was significantly superior to IMRT and VMAT in most of the relevant values for the bladder and femoral heads in the supine position. IMRT and VMAT yielded identical results, indicating that TOMO was superior to IMRT and VMAT in sparing OARs and planning quality parameters.

Acute gastrointestinal toxicity, such as diarrhea, is one of the most common complications of rectal chemoradiotherapy. Several studies have shown a strong dose-volume relationship between the irradiated small bowel volume and the severity of diarrheal toxicity at different dose levels [5, 18–20]. Previous studies have constructed predictive models for acute toxicity based on the strong dose-volume relationship between the irradiated small bowel volume and acute diarrhea at all dose levels [18]. Zhao et al. indicated that IMRT plans will lower the risk of acute toxicity to the small bowel after chemoradiotherapy [21]. However, our study revealed no differences in the risk of acute toxicity to the small bowel after chemoradiotherapy among TOMO, VMRT, and IMRT in both simulated positions.

A limitation of this study is that CT scan was the only imaging technique used to determine the tumor target. Other modern imaging modalities, such as PET, endoscopic ultrasound, and MRI, might increase the useful information. Previous studies have demonstrated that MRI or PET-CT matching can decrease inter-clinician variations, irrespective of the introduction guidelines [22, 23]. Another limitation of our study is the number of subjects used for research, although the optimal number required in such studies remains unknown. Recently, Fuller et al. reached similar results for target volume contouring in rectal irradiation with 17 observers and 4 patients [24].

In conclusion, our results demonstrated neither the prone nor the supine position yields superior values of OAR sparing. The TOMO technique was the best technique in the current study. Although TOMO, IMRT and VMAT achieved comparable target coverages, TOMO was superior in OAR sparing, except in the small bowel. Moreover, TOMO was superior to IMRT and VMAT in most clinically evaluated endpoints.

## MATERIALS AND METHODS

### Patient data and simulation

Patients with LARC were treated for primary tumors and regional lymph node metastases using methods approved by the multidisciplinary hepatogastrointestinal tumor board at Shuang Ho Hospital. Patients previously treated at our facility for rectal cancer or rectosigmoid colon cancer were chosen for this study. The patient inclusion criteria included an age range of 20 to 80 years, an Eastern Cooperative Oncology Group (ECOG) performance score of 0, 1 or 2, and pathology-proven rectal cancer or rectosigmoid colon cancer. Tumors were staged according to the 6th edition of the American Joint Committee on Cancer (AJCC) using the 2006 Criteria and the 7th edition of the AJCC using the 2010 Criteria. Positron emission tomography (PET) or whole-body computed tomography (CT) was performed to eliminate the existence of distant metastases. All procedures of patient acquisition followed the tenets of the Declaration of Helsinki and were approved by the Institutional Review Committee at Shuang Ho Hospital, Taipei Medical University.

Simulated non-contrast CT images, with oral fluid contrast to identify the small bowel were acquired with the patient in the supine position immobilized by pillow vacuum bags or in the prone position with belly broad fixation (Medtec and Sinmed Radiation Oncology Products, Orange City, IA, USA). The skin line marker was set at a slice thickness of 5 mm. The gross tumor volume (GTV) including the gross rectal or rectosigmoid tumor and positive pelvic lymph nodes was contoured by the physician based on the PET or initial CT image with contrast fusion image. The clinical target volume (CTV) was defined as the GTV plus 3-5 mm to the anterior, posterior, right and left directions and 5 cm into the superior and inferior regions. PTV margins were provided by the physician and varied from case to case. The prescription dose was 1.8 Gy x 28 fractions for a total dose of 50.4 Gy. OARs included the small bowel, urinary bladder and bilateral femoral head, over the same coverage of the CTV.

All plans aimed to achieve a minimum dose of greater than 97% and a maximum dose of less than 110% of the prescribed dose. The primary objectives with regard to the OARs were defined as follows: small bowel Dmax < 50 Gy and urinary bladder V48.07 < 25%, V44.87 < 35% and V34.98 < 50%. The above constraints were modified according to Quantitative Analyses of Normal Tissue Effects in the Clinic (QUANTEC) [14]: V75 < 50%; V70 < 35%; V65 < 50% based on the recommended prostate cancer treatment RTOG 0415 and a targeted dose of 78 Gy. The secondary objectives marked urinary bladder V30, V40, V50 and bilateral femoral head for statistical analysis. As a result of the tumor coverage requirements, a waiver could be applied for these dose constraints.

CTV coverage was evaluated using the conformity index (CI) and the heterogeneity index (HI), which were calculated as follows [15]:

$$CI=\frac{TV_{D}\times \;TV_{D}}{TV\times \;V_{D}}$$

TV_D_ is the target volume covered by the prescribed dose, TV is the target volume, and V_D_ is the volume enclosed by the prescribed isodose surface.

$$\text{HI}=\frac{D_{1}}{D_{95}}$$

D_1_ and D_95_ are, respectively, the doses encompassing 1% and 95% of the target volume.

### TOMO technique

Tomotherapy plans were calculated on the Tomotherapy Planning Station Hi-Art® Version 4.2.3 workstation (Tomotherapy Incorporated, Madison, WI, USA) with a superposition/convolution algorithm. Due to work station limitations, CT contouring and OARs images were drawn in Version 9.2 of the Pinnacle3 planning system and transferred to the TOMO planning system. All CT contours, whether supine or prone CT images, were planned by helical mode. A 6-MV photon beam was used. Similar coverage of the CTV to that of TOMO and VMAT was confirmed.

### VMAT technique

For treatment planning, images were acquired using a spiral CT scanner without contrast. The VMAT plans used 2 full arcs sharing the same isocenter with a 10-MV photon beam. The treatment protocols for the 20 patients treated with two full arcs were planned with start and stop angles of 180° and 181°, respectively, which were delivered with a counterclockwise rotation.

### IMRT technique

A 10-MV photon beam with six to seven co-planar beams and CT-based treatment planning (Pinnacle version 9.2) was used. The doses were delivered using a linear accelerator (LINAC) equipped with multi-leaf collimators (MLC). Similar coverage of the CTV to that of IMRT and VMAT was confirmed.

### Statistical analysis

Data were collected retrospectively from medical records, and 20 patients were included in this analysis. The differences in dosimetric parameters in the same simulated position were compared among the three planning techniques using Wilcoxon's signed-rank test: IMRT with VMAT, IMRT with TOMO, and VMAT with TOMO. A matched-pairs t-test was used to evaluate the dosimetric parameters in the different simulated positions between the 2 different planning techniques with 15 supine patients and 5 prone patients. Data analysis was performed using the Statistical Package for Social Sciences (SPSS) 20 (SPSS Inc., Chicago, IL). A p-value < 0.05 was considered statistically significant.
